# *In silico* Identification and Taxonomic Distribution of Plant Class C GH9 Endoglucanases

**DOI:** 10.3389/fpls.2016.01185

**Published:** 2016-08-12

**Authors:** Siddhartha Kundu, Rita Sharma

**Affiliations:** ^1^Department of Biochemistry, Dr. Baba Saheb Ambedkar Medical College & HospitalNew Delhi, India; ^2^Mathematical and Computational Biology, Information Technology Research Academy, Media Lab AsiaNew Delhi, India; ^3^School of Computational and Integrative Sciences, Jawaharlal Nehru UniversityNew Delhi, India

**Keywords:** artificial neural network, carbohydrate binding module, cellulose, endoglucanase, glycoside hydrolase, hidden markov models

## Abstract

The glycoside hydrolase 9 superfamily, mainly comprising the endoglucanases, is represented in all three domains of life. The current division of GH9 enzymes, into three subclasses, namely A, B, and C, is centered on parameters derived from sequence information alone. However, this classification is ambiguous, and is limited by the paralogous ancestry of classes B and C endoglucanases, and paucity of biochemical and structural data. Here, we extend this classification schema to putative GH9 endoglucanases present in green plants, with an emphasis on identifying novel members of the class C subset. These enzymes cleave the β(1 → 4) linkage between non-terminal adjacent D-glucopyranose residues, in both, amorphous and crystalline regions of cellulose. We utilized non redundant plant GH9 enzymes with characterized molecular data, as the training set to construct Hidden Markov Models (HMMs) and train an Artificial Neural Network (ANN). The parameters that were used for predicting dominant enzyme function, were derived from this training set, and subsequently refined on 147 sequences with available expression data. Our knowledge-based approach, can ascribe differential endoglucanase activity (A, B, or C) to a query sequence with high confidence, and was used to construct a local repository of class C GH9 endoglucanases (*GH*9*C* = 241) from 32 sequenced green plants.

## Introduction

Cellulose, a straight chain organic polymer of several hundreds of repeating disaccharide units of D-glucopyranose in a β(1 → 4) glycosidic linkage, is present in the primary cell wall of plants, algae, and oomycetes, and is also a critical component of bacterial biofilms (Updegraff, 1969; Yoshida Y. et al., 2006; Reardon-Robinson et al., 2014; Augimeri et al., 2015). Unlike the α(1 → 4) linked glucans of starch (coiled) and glycogen (branched), the β(1 → 4) bond of cellulose imposes several constraints on its structural conformation, rendering it, inflexible and stiff. Whilst, the bacterial forms are relatively uniform in constitution, plant cell walls are heterogenous, with a mixture of cellulose, hemicelluloses, and lignin (Klemm et al., 2005). The cohesive structure of cellulose is aided, additionally, by a rich network of non-covalent hydrogen bonds between the hydroxyl (−*OH*^−^) groups of the glucose moieties of its constituent microfibrils. The resultant macromolecule is stable, and can only be fragmented at elevated temperatures (>350°C) and pressure, in association with concentrated acids (Agarwal et al., 2012; Paulsen et al., 2014). The presence of cellulose in primary cell walls, whilst protective and strength conferring, is also important in the development and maintenance of bacterial biofilms for host interaction (Rhizobiaceae, Enterobacteriaceae, Acetobacteriaceae, etc.; Augimeri et al., 2015). This stability of cellulose, mandates a breakdown into constituent mono- and oligo-saccharides, prior to major patho-physiological events in plants. In fact, plant development, along with stress adaptor mechanisms, are critically dependent on the digestion of cellulose (del Campillo et al., 2012; Kundu, 2015a).

**Figure 1:**
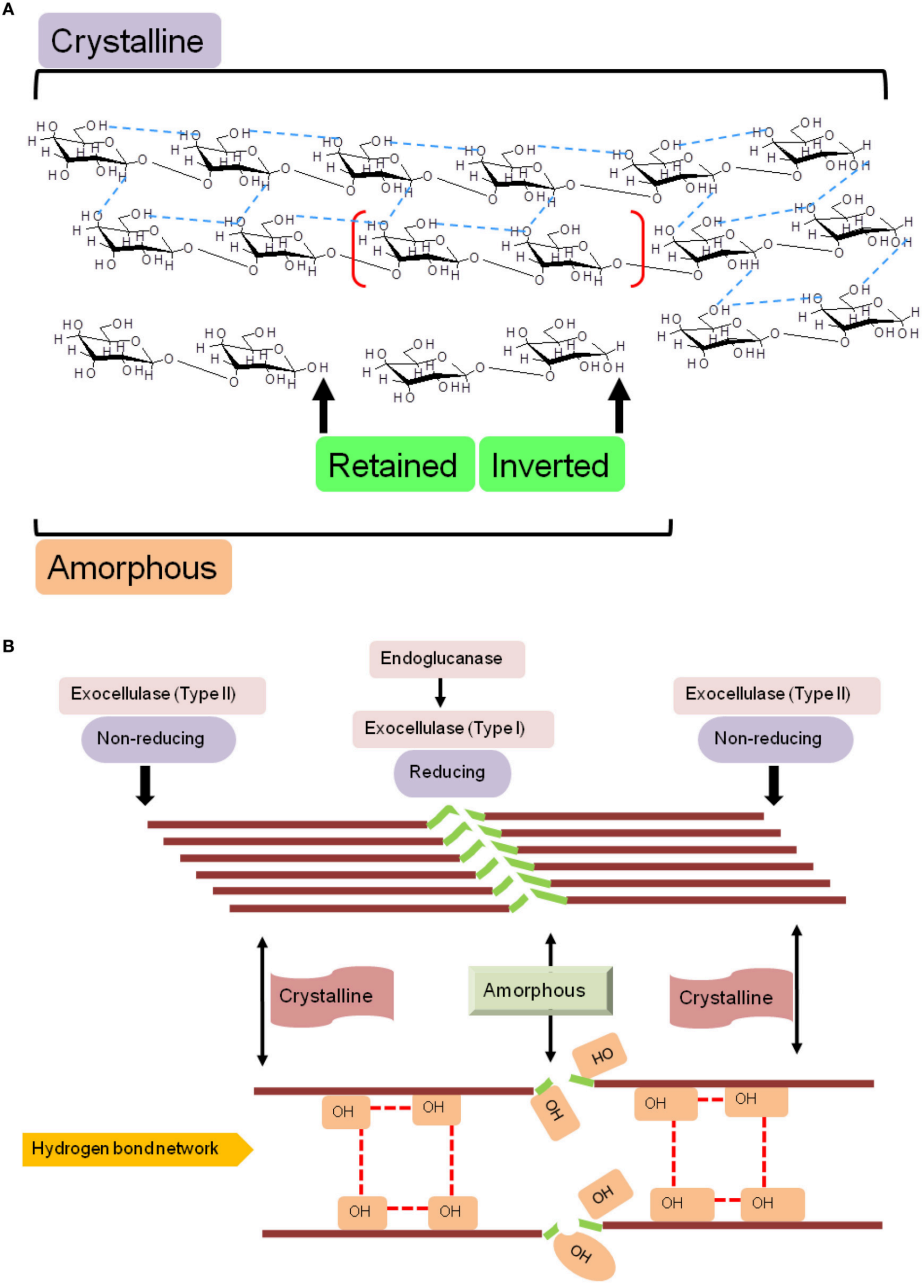
**Schematic diagram of the molecular structure of cellulose. (A)** Naturally occurring cellulose is a straight chain glucan, and is a mixture of crystalline and amorphous regions, a nomenclature based on the proportion of intermolecular (inter- and intra-strand) hydrogen bonds. Cellulases, may retain or invert the hydroxyl (OH^−^) on the anomeric carbon, a characteristic that is fold dependent, and **(B)** Structural and functional correlation of determinants of cellulose hydrolysis. The lack of a rigid structure (amorphous) renders cellulose amenable to catalytic cleavage by endoglucanase activity. Exocellulases (terminal), may act exclusively, but, usually effect digestion in association with endocellulases.

Endoglucanases, or cellulose hydrolases (EC3.2.1.4), cleave the β(1 → 4) glycosidic linkage of adjacent D-glucopyranose residues of the straight-chain glucan by introducing water molecules (Figure 1A). These enzymes comprise the superfamilies' GHs- 5–9, 12, 44, 45, 51, 74, and 124 (Lombard et al., 2014). The acid/base (A^+^/B^−^) catalytic mechanism of these enzymes to liberate mono- or oligo-saccharides, is facilitated by region as in endo- and exo-glucanses; and may exhibit chemical- (glucose, xylose, mannose, galactose, arabinose), and conformal-bias (retaining, inverting; Figure 1A and Table 1). Ancillary factors that contribute to substrate conversion include, the dominant secondary structural element (sse), presence and nature of the carbohydrate binding module(s), and the non-catalytic active site residues. The structural fold(s) for a retaining enzyme (A^+^/B^−^ = Glu/Glu) are (β/α)_8_ (GH5, 10, 26, 44, 51), and a β-jelly roll (GH7, 10). An inverting endoglucanase (A^+^/B^−^ = Asp/Asp or Asp/Glu), on the other hand, possesses the (α/α)_6_ (GH6, 8, 9, 45, 48, 124) or seven-fold β-propeller (GH7) folds.

**Table 1:** **Characteristics of GH9 endoglucanases associated with cellulase activity**.

**CAZy Family**	**Enzymatic activity (EC 3.2.1.x; EC 2.4.1.y)**	**Region (T/NT/TR/TN)**	**Mechanism (R/I)**	**Active site (A^+^/B^−^)**	**SSE**
5	*x* ∈{4, 8, 21, 25, 45, 58, 73, 74, 75, 78, 91, 104, 123, 132, 149, 151, 164, 168}	NT	R	(E/E)	(β/α)_8_
6	*x* ∈{4, 91}	TN	I	(D/D)	U
7	*x* ∈{4, 73, 132, 176}	TR	R	(E/E)	2(β_8_)
8	*x* ∈{4, 8, 73, 132, 156}	T;NT	I	(D/E)	(α/α)_6_
9	*x* ∈{4, 6, 21, 73, 74, 91, 151, 165}	NT	I	(D/E)	(α/α)_6_
12	*x* ∈{4, 73, 151}, *y* ∈{207}	NT	R	(E/E)	2(β_8_)
44	*x* ∈{4, 151}	NT	R	(E/E)	(β/α)_8_
45	*x* ∈{4}	NT	I	(D/D)	(α/α)_6_
48	*x* ∈{4, 14, 176}	T; NT	I	(U/E)	(α/α)_6_
51	*x* ∈{4, 8, 37, 55}	NT	R	(E/E)	(β/α)_8_
74	*x* ∈{4, 150, 151}	TR	I	(D/D)	7(β_4_)
124	*x* ∈{4}	NT	I	(U/U)	U

T, terminal; TR, terminal reducing (exo-); TN, terminal non-reducing (exo-); NT, non-terminal (endo-); R, Glucosyl retaining; I, Glucosyl inverting; A/B, residues for Acid/Base catalysis; D, Aspartic acid; E, Glutamic acid; U, Uncharacterized; SSE, secondary structural element; sfm, superfamily; SSEs: (β/α)8, 8-fold TIM (triosephosphate isomerase) barrel; (α/α)6, 6-fold barrel (Toroid); 7(β)4, 7-fold β-propeller; 2(β)8, β-jelly roll.

The carbohydrate binding module(s) (CBM) or cellulose binding domain(s) (CBD), present in these proteins dictate their association-dissociation kinetics with specific carbohydrate moieties and facilitate differential catalysis. These domains range from 40 (CBM1) to 200 amino acids (CBM17), and are present in several organisms (fungi, CBM1; bacteria, CBM2, 3, etc.; *D. discoideum*, CBM8; yeast, CBM54; plants, CBM49; Blume and Ennis, 1991; Koseki et al., 2008). The range of substrates bound include simple (galactose/lactose, CBM32; mannose, CBM13; chitin, CBM5, 12, 14, 18, 33; Newstead et al., 2005; Uni et al., 2009; Abramyan and Stajich, 2012; Li et al., 2015); compound (cellulose, CBM1-6, 8-11, 13, etc.; glycogen, CBM21, 48; starch, CBM20, 25; xylans, CBM22; polygalactouronic acid, CBM32; Abbott et al., 2007; Palomo et al., 2009; Janecek et al., 2011); and complex (lipopolysaccharide/lipoteichoic acid, CBM39; LacNAc; Bachman and McClay, 1996; Ficko-Blean and Boraston, 2006) molecules. Whilst, the *de facto* biological role for proteins with these domains is catalytic, there is ample evidence for the contrary (CBM1, 29, 43; Barral et al., 2005; Yoshida et al., 2005; Obembe et al., 2007). The cellulosome, is a complex of enzymes that participates in the assembly-disassembly of cellulose, along with several critical ancillary molecules (primer), and essential co-factors (Peng et al., 2002; Mansoori et al., 2014). This functional structure, along with molecular networks of co-expressed glycoside hydrolases is driven by the non-specific interactions of CBMs, either alone or in tandem with other enzyme-specific domains (Peng et al., 2002; Sharma et al., 2013; Mansoori et al., 2014). Perhaps the most intriguing constraint imposed by these domains is that of differential catalysis, i.e., same substrate, variable regions, and different enzymes. Naturally occurring cellulose is composed of at least two mutually exclusive regions: crystalline and amorphous (Figure 1). The inter- and intra-strand network of hydrogen bonds renders these microcrystalline regions well ordered, a feature that imposes an upper bound on the binding capacity of endoglucanases (CBM9, 49). In contrast, the latter, lacks this organization, permitting a far greater number of enzyme binding sites (CBM4, 6, 17, 28; Boraston et al., 2003; Jamal et al., 2004; Alahuhta et al., 2010). There are several hypotheses over the role of these domains in catalysis. These include, a physical, fix-and-stretch mechanism of the carbohydrate moiety from its parent glucan. This notion is based on the abundance of aromatic amino acids (W/F/Y) in these modules (Simpson et al., 2000; Roske et al., 2004; Flint et al., 2005; Tunnicliffe et al., 2005), and the presence of calcium (CBM35, 36, 60; Montanier et al., 2010).

Plant GH9 endoglucanases, like other members possess an activity profile that includes endoglucanase (EC3.2.1.4), lichenase (EC3.2.1.6, CBM4, 6, 13, 32, 54), mixed endoglucanase (EC3.2.1.73), exoglucanase (EC3.2.1.74, CBM2, 6, 10), cellobiohydrolase (EC3.2.1.91, CBM1-5, 10), and endo-xyloglucanase (EC3.2.1.151, CBM1-3, 30, 35, 44; Figure 1B) activity. The associated CBMs however, are not, always present together in a single sequence (Lombard et al., 2014). Extant classification schema into classes A, B, and C, are centered on sequence similarity, codon usage, distribution of intron-exon boundaries, and presence/absence of a trans-membrane domain (TM, class A) or secretory peptide (SP, class B; Mølhøj et al., 2002; Libertini et al., 2004). Whilst, the association between function and these indices is reasonably predictive, the similarity between sequences of classes B and C could potentially vitiate simpler clustering protocols. A sequence was identified in the class B cellulase subfamily (SlCel9C1; *S. lycopersicum*) which possessed a novel domain that was designated as CBM49 (IPR019028, PF09478; Urbanowicz et al., 2007a). This module is similar to the CBM2 in *C. filmi*, and implies, that this subset of enzymes could potentially function as a general purpose plant cellulase with catalysis of both, ordered and amorphous regions (class C activity; McLean et al., 2000; Boraston et al., 2004; Zhang et al., 2015). However, *in vitro* and *in vivo* experiments by these and other investigators, with the mature protein, suggest that this domain is removed prior to mature transcript formation, accounting for the refractoriness of this class to crystalline cellulose as a substrate *in vitro* (Urbanowicz et al., 2007a).

The role of GH9 enzymes in regulating plant physiology is unequivocal. The presence of the TM-domain localizes class A GH9 endoglucanases to the cell wall (primary, secondary), and associated structures (cell plate), thereby, dictating assembly by *de novo* cellulose biosynthesis in these regions (Nicol et al., 1998; Zuo et al., 2000; Sato et al., 2001; Mølhøj et al., 2002; Mansoori et al., 2014; Yu et al., 2014). Similarly, extracellular secretion, suggests a distributed influence and may facilitate a rapid response to stress (abiotic, biotic) by classes B and C enzymes. Class C GH9 endoglucanses, too, can influence development and response to stress), modify biofilm development for symbiotic or bacterial interactions, and can facilitate direct biomass conversion. Whilst, the high proportion of crystalline cellulose in the primary cell wall can be effectively and rapidly hydrolyzed (Urbanowicz et al., 2007a); its absence in the cell walls of root hair cells and endosperm, has also been attributed to active inhibition by this class of enzymes (AtGH9C1; *A. thaliana*; Shpigel et al., 1998; Sturcova et al., 2004; Otegui, 2007; del Campillo et al., 2012).

Complex polysaccharides, involving cellulose, are critical for host-bacterial interactions, and are secreted by the infecting bacteria, or activated in the host (plant roots/root hair, intestinal and lung epithelia; Cannon and Anderson, 1991; Mathee et al., 1999; Zogaj et al., 2001; White et al., 2003). The biofilms, thus formed facilitate aggregation, permit intercellular transfer of critical nutrients and signaling molecules, and can confer additional features (antibiotic resistance) by genetic exchange. A dual role for class C GH9 endoglucanases has been postulated and experimentally demonstrated in: (a) assisting infection, formation, and release into legume nodules by *Rhizobium* spp. (CelC2; Robledo et al., 2012), and (b) colonization by *Rhizobium* spp. and *A. tumefaciens*, by maturation/branching of this extracellular matrix (Matthysse et al., 1995; Robledo et al., 2012). The localization of AtGH9C1 in root hair cells (del Campillo et al., 2012), and concomitant infection with *A. tumefaciens* could increase the bacterial load (Matthysse et al., 2005), thereby, enhance the tumor forming capacity of these gram negative bacteria. Here, a combination of hydrolysis, translocation, and elongation-by-branching of cellulose by class C GH9 endoglucanases (plant, bacterial), would ensure optimal colonization. The most exciting role for class C GH9 endoglucanases, is their potential contribution to the biofuel industry (Lopez-Casado et al., 2008). Cellulose digestion can be mediated by the simultaneous presence of endo- and exo-glucanase regions in a single protein (CelA; *C. bescii*), a combinatorial association of endo- and exoglucanases in the cellulosome (CclEXL-1; *C. clariflavum*), and the possession of specialized modules (CBM49, CBM2) (Urbanowicz et al., 2007a; Chung et al., 2015; Artzi et al., 2016).

The methods and algorithms used to classify enzymes (superfamily, family, subfamily) depend on sequence based features or the conformational mapping of 3D information (secondary/tertiary/quaternary) to the primary structure. Supervised learning, is a machine learning method, that mandates training of an algorithm on well defined sets, and includes support vector machines (SVMs), regression analysis, neural networks, among several others. The SVM algorithm creates a hyperplane, and seeks to identify data points closest (support vectors) to this. It also entails an optimization to maximize the inter planar spacing. SVMs, for protein sequence classification will typically consider combinatorial associations of the amino acids sequence, such as pairs and triplets, etc. from the set of training sequences for feature extraction. HMMs, on the hand are models of a multiple sequence alignment, and represents a consensus of all the columns selected. SVMs, despite their predictive propensity, require unambiguous data and draw upon results from multiple rounds of pairwise comparisons (multiclass SVMs). Further, sequences with high sequence identity/similarity and common catalytic function (subfamily), might be better candidates for classification by SVM schema. GH9 endoglucanases have an intermediate level of sequence identity/similarity, with dominant function being clearly attributed to the presence of definitive region(s) in the N- (secretory peptide, transmembrane domain) and C-termini (CBM49) of the mature protein, and their linkage to the remainder of the protein (Mølhøj et al., 2002; Libertini et al., 2004; Urbanowicz et al., 2007a). The aforementioned enzyme specific constraints, and a superior performance assessment of HMMs over SVMs, lends credence to our choice of HMM-ANN as the analytic platform to stratify GH9 endoglucanases (Khater and Mohanty, 2015).

There are a number of general Hidden Markov Model based predictors of protein function and classification (Gene3D, Pfam; Sonnhammer et al., 1998; Lees et al., 2010). These methods, despite providing initial pointers to novel candidate sequences, are unable to segregate closely related proteins. This limitation may be compensated, in-part, by populating the training dataset with sequences that meet stringent criteria, such as the availability of empirical data (Kundu, 2012). Alternate possibilities include, the use of pre-defined thresholds for data output, methods to screen the HMM output, and defining numerical patterns of domain dominance. In this study, we use a reverse look-up strategy to infer plant cellulose hydrolysis activity of putative sequences, from proteins which have been previously characterized. Since, our objective was to scan this data for highly probable class C sequences, a mathematical filter was developed to screen these on the basis of the HMM scores of the included profiles. Rigor of the prediction schema was ensured by formulating and validating indices to ascertain functionality from the returned results. Since the rules governing this association are complex, an ANN based-clustering protocol was chosen to infer and later, predict class assignment. The product of ANN-predicted values (weights, modifiers, constants) with one or more variables may be used to approximate a given function. This subset of supervised machine learning methods is non-linear and mandates the presence of high-confidence training datasets, but is able to delineate novel patterns with reasonable accuracy, and is suitably robust.

## Methods

### Dataset creation

**Figure 2:**
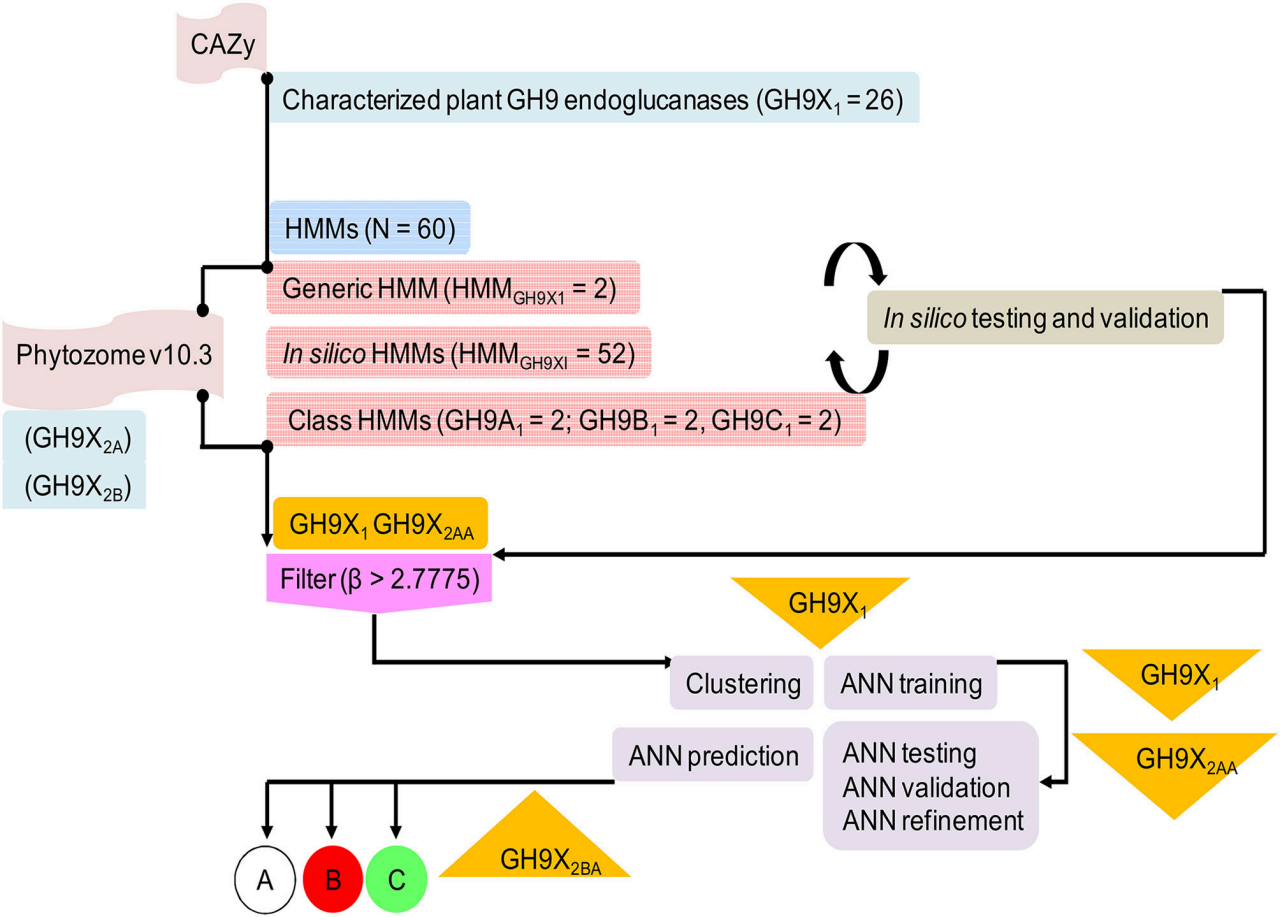
**Overview of the protocol adopted in this work describing sequence downloads, model building, and analysis**. The information flow depicted was adhered to ensure high quality readouts at each sublevel.

We downloaded GH9 endoglucanse protein sequences from the CAZy (http://www.cazy.org) and UniProtKB (http://uniprot.org) databases (Figure 2). All associated information such as domain architecture, presence of specialized motifs, reaction chemistry, and physiological roles were cross referenced with InterPro (https://www.ebi.ac.uk/interpro), Pfam (http://pfam.xfam.org), SMART (http://smart.embl-heidelberg.de), and PROSITE (http://prosite.expasy.org) databases. Only endoglucanases with (a) demonstrable cellulase activity (EC 3.2.1.x), (b) transcript data with biochemical and/or physiological function, and (c) an available 3D structure, were short listed. These sequences were clustered in accordance with previously defined criteria into classes A, B, and C cellulases (Sonnhammer et al., 1998; Apweiler et al., 2000; Letunic et al., 2002; Sigrist et al., 2010; Lombard et al., 2014). The 3D models of the submitted sequences were received from the Phyre2 server (Kelley et al., 2015). This preliminary compilation of 157 sequences was filtered, to exclude redundant data, such that the final dataset of GH9 sequences (*GH*9*X*_1_ = 26 = {*GH*9*A*, *GH*9*B*, *GH*9*C*}) spanned 11 distinct plant genera (Table S1). Further partitioning, was based on the availability of enzyme specific experimental data (*GH*9*A*_1_ = 6; *GH*9*B*_1_ = 16; *GH*9*C*_1_ = 4; Table S1). These sequences were used to construct the HMMs and train the ANN (Table 2 and Table S2). Similarly, the sequences used to test these methods were divided into two groups: *GH*9*X*_2*A*_ = 147 (available expression data), and *GH*9*X*_2*B*_ = 874 (curated primary transcript data from the eukaryotic subclade Viridiplantae). The protein sequences for these datasets were downloaded from the Phytozome v10.3 (http://phytozome.jgi.doe.gov/pz/portal.html) database, and were mutually exclusive (*GH*9*X*_1_ ∩ *GH*9*X*_2_ = 0). *GH*9*X*_2*A*_, comprised sequences from *Arabidopsis thaliana* (*GH*9*X*_2*A_atha*_ = 24), *Glycine max* (*GH*9*X*_2*A_gmax*_ = 39), *Oryza sativa* (*GH*9*X*_2*A_osat*_ = 25), *Solanum lycopersicum* (*GH*9*X*_2*A_slyc*_ = 21), and *Zea mays* (*GH*9*X*_2*A_zmay*_ = 38) (Table S3A). Since, the sequences in the training set were few, *GH*9*X*_2*A*_, was used to refine and partially validate the HMM and ANN predictions, as well as, corroborate their function *in vivo* (Zimmermann et al., 2004; Rensink et al., 2005; Skibbe et al., 2006; Cao et al., 2012).

**Table 2:** **Summary and select details of HMM profiles utilized in this work**.

	**Profile**	**Sequences**		**AL**	**ML**	**ES**	**RP**
HMM_GH9X_ = 2	E5Tx1D	26		754	520	0.8	0.592
	E5Tx3D			825	449	0.79	0.589
HMM_GH9A_ = 2	E4TA1D	6		622	618	0.42	0.592
	E4TA3D			615	495	0.5	0.592
HMM_GH9B_ = 2	E4TB1D	16		534	501	0.64	0.589
	E4TB3D			574	434	0.6	0.592
HMM_GH9C_ = 2	E4TC1D	4		651	625	0.48	0.589
	E4TC3D			622	566	0.53	0.592
	E5Tx3D			825	449	0.79	0.589
		**Training**	**Validation (UID)**				
HMM_GH9AI_ = 12	TAE11D	5	1 (Q6X680)	622	618	0.42	0.594
	TAE13D			600	511	0.48	0.588
	TAE21D	5	1 (O04890)	622	619	0.42	0.596
	TAE23D			613	503	0.49	0.588
	TAE31D	5	1 (G0ZTA3)	622	619	0.41	0.587
	TAE33D			615	490	0.47	0.587
	TAE41D	5	1 (Q6DMM4)	622	618	0.42	0.591
	TAE43D			606	499	0.48	0.594
	TAE51D	5	1 (D3JWK8)	622	618	0.42	0.592
	TAE53D			619	493	0.49	0.591
	TAE61D	5	1 (Q38890)	619	619	0.42	0.588
	TAE63D			590	511	0.47	0.585
HMM_GH9BI_ = 32	TBE11D	15	1 (Q42872)	550	490	0.62	0.589
	TBE13D			529	437	0.6	0.589
	TBE21D	15	1 (O04972)	556	490	0.62	0.588
	TBE23D			554	442	0.6	0.593
	TBE31D	15	1 (Q41012)	546	490	0.62	0.59
	TBE33D			554	437	0.6	0.592
	TBE41D	15	1 (Q93WZ0)	547	492	0.63	0.592
	TBE43D			554	437	0.6	0.589
	TBE51D	15	1 (Q93WZ1)	539	494	0.63	0.592
	TBE53D			560	437	0.6	0.591
	TBE61D	15	1 (P22503)	537	496	0.61	0.588
	TBE63D			552	437	0.59	0.593
	TBE71D	15	1 (Q40763)	556	490	0.62	0.589
	TBE73D			552	434	0.6	0.592
	TBE81D	15	1 (Q9XIY8)	537	495	0.63	0.589
	TBE83D			566	435	0.6	0.591
	TBE91D	15	1 (Q6DMM3)	553	490	0.62	0.591
	TBE93D			564	438	0.6	0.59
	TBE101D	15	1 (P94114)	537	494	0.63	0.592
	TBE103D			563	439	0.6	0.588
	TBE111D	15	1 (Q42875)	534	490	0.62	0.59
	TBE113D			560	434	0.59	0.588
	TBE121D	15	1 (Q42871)	528	489	0.61	0.591
	TBE123D			555	436	0.58	0.587
	TBE131D	15	1 (O82473)	552	492	0.62	0.589
	TBE133D			556	440	0.6	0.587
	TBE141D	15	1 (Q9CAC1)	544	490	0.62	0.587
	TBE143D			558	434	0.6	0.592
	TBE151D	15	1 (Q9SRX3)	545	492	0.62	0.588
	TBE153D			555	437	0.6	0.589
	TBE161D	15	1 (Q9ZTL0)	543	493	0.63	0.593
	TBE163D			560	434	0.59	0.587
HMM_GH9CI_ = 8	TCE11D	3	1 (Q8LJP6)	648	625	0.47	0.592
	TCE13D			609	579	0.5	0.588
	TCE21D	3	1 (Q5NAT0)	636	621	0.43	0.588
	TCE23D			605	575	0.47	0.59
	TCE31D	3	1 (Q93WY9)	647	621	0.47	0.589
	TCE33D			626	557	0.51	0.588
	TCE41D	3	1 (Q9ZSP9)	642	625	0.47	0.59
	TCE43D			612	566	0.52	0.592

UID, Uniprot Identifier; HMM_GH9X_, Dominant function of sequence (X = A, B, C); Text S1 and S3 in Supplementary Material; HMM_GH9XI_, Dominant function of sequence for in silico abalysis (X = A, B, C); Text S2 in Supplementary Material; AL, Alignment Length; ML, Model Length; ES, Effective number of Sequences; RP, Relative Entropy per Position.

### Construction of a profile database

We used the sequences of the training dataset (*GH*9*X*_1_) to construct the HMMs (*HMM*_*GH*9*X*_ = 60) (Table 2; Text S1–S3), and broadly segregated into sequence- (*HMM*_1*D*_ = 30) and their corresponding structure-based (*HMM*_3*D*_ = 30) profiles. Since, a 3D- alignment is based on the conformational arrangement of secondary structural elements and active site residues, a higher correlation to function, of the corresponding HMM was subsumed. Alignments and cladograms for each dataset were generated separately with the STRAP (Structural Alignment of Proteins; http://www.bioinformatics.org/strap) and Clustal Omega v1.2.1suite of programs, format conversion was server-based (http://www.ibi.vu.nl/programs/convertalignwww), and HMMER 3.0 (http://hmmer.janelia.org) was used for model building, analysis, database construction, and similarity studies with the input sequences (Gille et al., 2014).

The highest scoring region/subdomain, for each HMM profile, of a sequence was considered for analysis (Table 2).

The large number of profiles (30 pairs = 1D and 3D) were utilized to: (a) query the putative proteome of 34 green plants and algae present in Phytozome v10.3, for putative GH9 endoglucanase homologs (*HMM*_*GH*9*X*_; 1 pair), (b) ascertain the profile decomposition of each test sequence (3 pairs; Enzyme activity of sequence : $\;={\bar{HMM}}_{GH9A},\;{\bar{HMM}}_{GH9B},\;{\bar{HMM}}_{GH9C}),$ and (c) compensate, for the reduced size of the training sequences, by deploying an exhaustive leave-out-one strategy to compute, analyze, and computationally validate, the profile HMM scores for each training sequence *HMM*_*GH*9*XI*_ = 26 pairs = *HMM*_*GH*9*AI*_ + *HMM*_*GH*9*BI*_ + *HMM*_*GH*9*CI*_) (Figure 2, Table 2; Table S2A). Here, every sequence was assumed, *a priori*, to possess dual membership, i.e., it was part of both the training and validation subsets for a particular profile. The raw HMM scores of the selected profiles of only those sequences that were used for validation, were considered and averaged (1D, 3D) This can be generalized as:

$$GH9XI=\{\begin{array}{l} \left(HMM_{GH9AI}\;-\;(\left(2\right)\left(GH9A_{1}-1\right))\right),\\ \;(HMM_{GH9BI}),\left(HMM_{GH9CI}\right),X=A\;Def.1\\ \left(HMM_{GH9AI}\right),(HMM_{GH9BI}\;-\;(\left(2\right)\left(GH9B_{1}-1\right))),\\ \;\left(HMM_{GH9CI}\right),X=B\;Def.2\\ \left(HMM_{GH9AI}\right),(HMM_{GH9BI}),(HMM_{GH9CI}\;-\;((2)\\ \;(GH9C_{1}-1))),X=C\;Def.3 \end{array}$$

Consider the following example. Since, the number of training sequences with class C activity are only four (*GH*9*C*_1_ = 4; number of class C profiles = 8), in the leave-one-out schema, only data from this single class C sequence (number of class C profiles = 2) was deemed relevant. Similarly, for this particular sequence all class A and B profiles scores would be taken into account (class A profiles = 12; class B profiles = 32). Thus, the combined HMM scores of these relevant profiles were considered (12 + 32 + 2 = 46 or 23 pairs), for this class C sequence (Table 2, Tables S2A,B).

### Screening filter

Profile HMMs (pHMMs), whilst being theoretically well grounded, do not offer unambiguous predictions, i.e., the query sequence is a function of the included profiles. Although, the resultant data may be filtered with the use of inclusion and threshold scores, an inter-profile comparison of scores with defined exclusion criteria is clearly desirable. Populating the ANN input with sequences with well-spaced HMM scores, can be accomplished by progressively screening out sequences which do not comply with these conditions. This filter compares the raw HMM scores of the constituent profiles, and outputs a quality score (β; Equation 1). The method is based on computing a modified Z-score of pairs of groups of profiles that comprise a sequence, i.e., a mixture of classes A, B, and C, calculated as $\left(C\left(3,2\right)=\left(\begin{array}{l} 3\\ 2 \end{array}\right)=3\right).$ Here,

$$\begin{array}{l} Group12:\;=\{\left({\bar{HMM}}_{GH9AI},{\bar{HMM}}_{GH9BI}\right),\;\\ \;\left({\bar{HMM}}_{GH9BI},{\bar{HMM}}_{GH9CI}\right)\}\;Def.4 \end{array}$$

$$\begin{array}{l} Group23:=\{\left({\bar{HMM}}_{GH9BI},{\bar{HMM}}_{GH9CI}\right),\;\;\\ \;\left({\bar{HMM}}_{GH9AI},{\bar{HMM}}_{GH9CI}\right)\}\;Def.5 \end{array}$$

$$\begin{array}{l} Group13:\;=\{\left({\bar{HMM}}_{GH9AI},{\bar{HMM}}_{GH9BI}\right),\;\\ \;\left({\bar{HMM}}_{GH9AI},{\bar{HMM}}_{GH9BI}\right)\}\;Def.6 \end{array}$$

(1) $$\beta =1/2\;(\sum_{i\;=\;1}^{i\;=\;3}\sum_{j\;=\;1}^{j\;=\;3}\alpha_{ij})\;\forall \;i\neq j,\;\alpha_{ij}=\alpha_{ji}$$

(2) $$\begin{array}{l} \alpha_{ij}=gp_{ij}=\left(|\mu_{i}-\mu_{j}|/100\right)\\ \;\left(|\mu_{i}-\mu_{j}|/\left(\sqrt[{2}]{\left(\sigma_{i}^{2}+\sigma_{j}^{2}/\tau \right)}\right)\right) \end{array}$$

$$\begin{array}{l} i,j\in \{1,2,3\}with\;i\neq j\\ \;\tau =2\;(members\;in\;each\;group)\\ \;\mu :\;=intra\;-\;group\;mean\\ \;\sigma^{2}:\;=intra\;-\;group\;variance\\ \text{ Z}:\;=inter\;-\;group\;z-score \end{array}$$

The final selection of the threshold value was a numerical refinement of min(β) of *GH*9*X*_1_ on *GH*9*X*_2*A*_ (**Figures 4A,B**), and its subsumed correspondence with the inter profile HMM difference (min(β) ↦ *median*(Δ*HMM*); *Equation* 8) (Tables S2C, S3C).

### ANN based assignment of dominant enzymatic activity of GH9 endoglucanases

As highlighted *vide supra*, confirmation of enzymatic activity can be unequivocally resolved only in a laboratory setting. Models, at best, offer the probability of a particular outcome. This measure of uncertainty is compounded by pHMM-based analytics and the paucity of experimental data. The native profile HMM scores *(P)* of a query sequence is one measure of ascertaining the function of a putative protein, i.e., max *(P)*. However, the proximity of these scores, especially in classes B and C sequences, precludes confidence in any such assignment. Descriptive statistics of these pairs-of-pairs means (α_12_, Group 12; α_23_, Group 23; α_13_, Group 13), suggests that this modification of the Z-score (Equations 2, 6-8), may provide a rigorous framework that could not only exclude sequences with equivalent profile HMM scores, while at the same time be used (β; Equation 1) to cluster sequences.

Cluster analysis (k-means), of the β-values of each sequence of *GH*9*X*_1_, was used to compute class-specific cluster means $\left(k=3;{\beta^{\prime }}_{A},{\beta^{\prime }}_{B},\beta_{C}^{\prime }\right)$ which were then graphed and plotted using a cluster-dendrogram (**Figures 4C,D**, Text S4). This value was chosen so as to maximize the distance between the centroids of the clusters, thereby, ensuring high confidence in the assignments (max(*between*_*SS*_/*total*_*SS*_)). Outliers, were removed to ensure rigor (**Figure 4D**). These β′- values were assumed to be linear combination of the weighted derived scores for each sub-group $\left(\beta^{\prime }≅\underset{i\;=\;1,2;j\;=\;2,3}{\sum }\left(\gamma_{ij}\right)\left(\alpha_{ij}\right),\forall \;i\neq j\right)$ of a particular sequence. The values of these weights (Text S5), and their confidence at 0.95 (1 − α|α = 0.05) were computed using an ANN (Hidden layers = 10; threshold = 0.01). The predicted ANN values (β″) in this leave-one-out (*GH*9*X*_1*A*_ = 24) approach, were then compared with the previously computed cluster means (β″ ≅ β′).

The absence of confirmatory enzyme kinetic data for the test sequences, i.e., mRNA (*GH*9*X*_2*A*_) and genomic-hypothetical proteins (*GH*9*X*_2*B*_), precludes the direct usage of cluster means (β′) or their approximations (β″), as unambiguous predictors of enzyme function. Here, instead, it was reasoned that an enzyme specific class interval, rather than a single value, for the HMM-ANN prediction on $GH9X_{2A}\;(\min\left(\beta \prime \prime_{GH9A}\right)\;\leq \;\beta \prime \prime_{GH9A}\leq \max\left(\beta \prime \prime_{GH9A}\right);\;\min\left(\beta \prime \prime_{GH9B}\right)\leq \beta \prime \prime_{GH9B}\leq \max\left(\beta \prime \prime_{GH9B}\right);\;\min\left(\beta \prime \prime_{GH9C}\right)\leq \beta \prime \prime_{GH9C}\leq \max\left(\beta \prime \prime_{GH9C}\right))$ for each enzyme function, along with select patterns of the computed α_*ij*_-values, may encompass function more effectively. The R-scripts (R-3.0.0) needed to analyze this data, and perform other miscellaneous tasks were coded in-house, or downloaded as packages. This included the ANNs (neuralnet), clustering, and plotting (cluster; fpc). Chemical structures were drawn using the ChemSketch suite (freeware) installed locally.

### Validating the integrated pipeline

The exhaustive leave-one-out strategy utilized for the computations, also functioned to cross validate (LOOCV) the predictions by the HMMs and the ANN, and was chosen to compensate for the paucity of training sequences. The criteria to validate, for selecting the appropriate HMM, was the equivalence of the highest scoring profile of a sequence with predicted enzyme function (Enzyme activity of a sequence = max (${\bar{HMM}}_{GH9A}$, ${\bar{HMM}}_{GH9B}$, ${\bar{HMM}}_{GH9C}$))., as a generalization for the analytic and *in silico* steps, as under:

$$GH9A:={\bar{HMM}}_{GH9A}>\left\{{\bar{HMM}}_{GH9B},{\bar{HMM}}_{GH9C}\right\}\;Def.7$$

$$GH9B:={\bar{HMM}}_{GH9B}>\left\{{\bar{HMM}}_{GH9A},{\bar{HMM}}_{GH9C}\right\}\;Def.8$$

$$GH9C:={\bar{HMM}}_{GH9C}>\left\{{\bar{HMM}}_{GH9A},{\bar{HMM}}_{GH9B}\right\}\;Def.9$$

$$\begin{array}{l} GH9AI\;:=\left(HMM_{GH9AI}\;-\;\left(\left(2\right)\left(GH9A_{1}\;-\;1\right)\right)\right)\\ \;>\{(HMM_{GH9BI}),(HMM_{GH9CI})\}\;Def.10 \end{array}$$

$$\begin{array}{l} GH9BI:\;=(HMM_{GH9BI}\;-((2)\left(GH9B_{1}\;-\;1\right)))\\ \;>\left\{\left({\bar{HMM}}_{GH9AI}),({\bar{HMM}}_{GH9CI}\right)\right\}\;Def.11 \end{array}$$

$$\begin{array}{l} GH9CI\;:=\left(HMM_{GH9C_{1}}\;-\;\left(\left(2)(GH9C_{1}\;-\;1\right)\right)\right)\\ \;>\left\{({\bar{HMM}}_{GH9AI}),\left({\bar{HMM}}_{GH9BI}\right)\right\}\;Def.12 \end{array}$$

Similarly, the index of measurement, chosen, to ascertain relevance of the ANN predicted values (β″) was based on the following:

(3) $$\beta \prime \prime \;≅\;\beta^{\prime }$$

(4) $$∵\beta^{\prime }\equiv \;max\;({\bar{HMM}}_{GH9A},{\bar{HMM}}_{GH9B},{\bar{HMM}}_{GH9C})$$

(5) $$∴\beta \prime \prime \equiv \;max\;({\bar{HMM}}_{GH9A},{\bar{HMM}}_{GH9B},{\bar{HMM}}_{GH9C})$$

The chi-squared (χ^2^) statistic was used to compare the two sets of numerical data points for *GH*9*X*_1_ (Equation 3). Since, these values were based on a restricted dataset, the procedure was repeated on *GH*9*X*_2*A*_. However, despite the availability of expression data for these sequences, information on the catalytic activity of their encoded proteins is undefined, and therefore, at best inferred (Equation 5).

### Analysis of biological significance of the ANN-based predictions using transcriptomic data

The relevance of these predictions was assessed using available gene expression datasets. For *O. sativa* and *A. thaliana*, extensive expression data for the anatomical and developmental stages is publically available. These were analyzed to observe the fluctuations in gene expression of some of the sequences identified in dataset (*GH*9*X*_2*A*_). The metadata for gene expression analysis in rice was downloaded from the rice oligonucleotide array database (ROAD; http://www.ricearray.org; Table S6A), whereas, the same for *A. thaliana* was extracted using GENEVESTIGATOR (https://genevestigator.com/gv; Table S6B; Zimmermann et al., 2004; Cao et al., 2012).

## Results

### Salient features of models and *in silico* analysis

**Figure 3:**
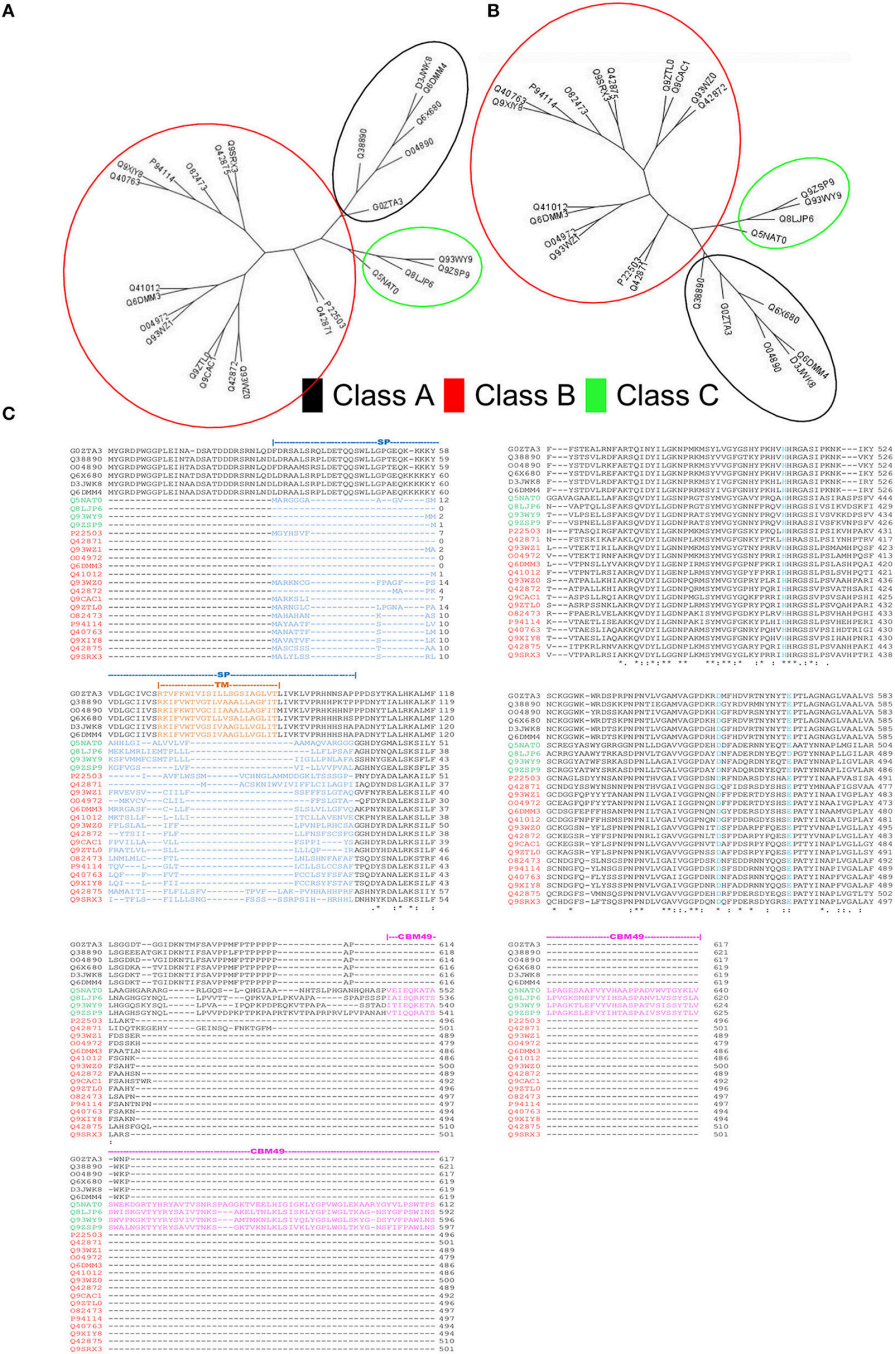
**Rationale for computational strategies deployed in this work. (A,B)** Comparison of the unrooted cladograms of sequence- (1D) and structure- (3D) based alignments results in a congruent grouping of sequences, a key assumption in the computation of average pHMM-scores. **(C)** Multiple sequence alignment of the sequences present in the training set. Whilst, class A sequences are characterized by a well defined transmembrane domain (TM), classes B and C possess a signal peptide sequence at their N-terminal ends. The carbohydrate binding module 49 (CBM49), defines presence of class C activity, and is present at the C-terminal and is ≈100–120 AA.

HMMs, with sequence and domain threshold values (*E*_*seq*_ = *E*_*d*_ = 10*E* − 06), were generated from the formatted alignments of amino-acid sequences and their corresponding modeled 3D-coordinates (Figures 2, 3). Since, the phylogenetic clades were similar for both sets of profiles (Figures 3A,B), profile pairs were averaged:

$$HMM_{GH9A}\;:\;=\;average\;(HMM_{GH9A1D},HMM_{GH9A3D})\;\left(Def.13\right)$$

$$HMM_{GH9B}\;:\;=\;average\;(HMM_{GH9B1D},HMM_{GH9B3D})\;\left(Def.14\right)$$

$$HMM_{GH9C}\;:\;=\;average\;(HMM_{GH9C1D},HMM_{GH9C3D})\;\left(Def.15\right)$$

The model(s) characteristics and summary are presented (Table 2). A fraction of the data (δ_*A*_ = *HMM*_*GH*9*AI*_/*HMM*_*GH*9*XI*_; δ_*B*_ = *HMM*_*GH*9*BI*_/*HMM*_*GH*9*XI*_; δ_*C*_ = *HMM*_*GH*9*CI*_/*HMM*_*GH*9*XI*_) was used for computing the profile scores (Tables S2A,B). Thus, for any class A sequence (*HMM*_*GH*9*AI*_ = 42, δ_*A*_ ≅ 0.72; *Def*.7). The equivalent data for any class B (*HMM*_*GH*9*BI*_ = 22, δ_*B*_ ≅ 0.38; *Def*.8), and C (*HMM*_*GH*9*CI*_ = 46, δ_*C*_ ≅ 0.79; *Def*.9) sequence was computed similarly (Tables S2A,B).

### Numerical determination and relevance of the filter for sequence selection

**Figure 4:**
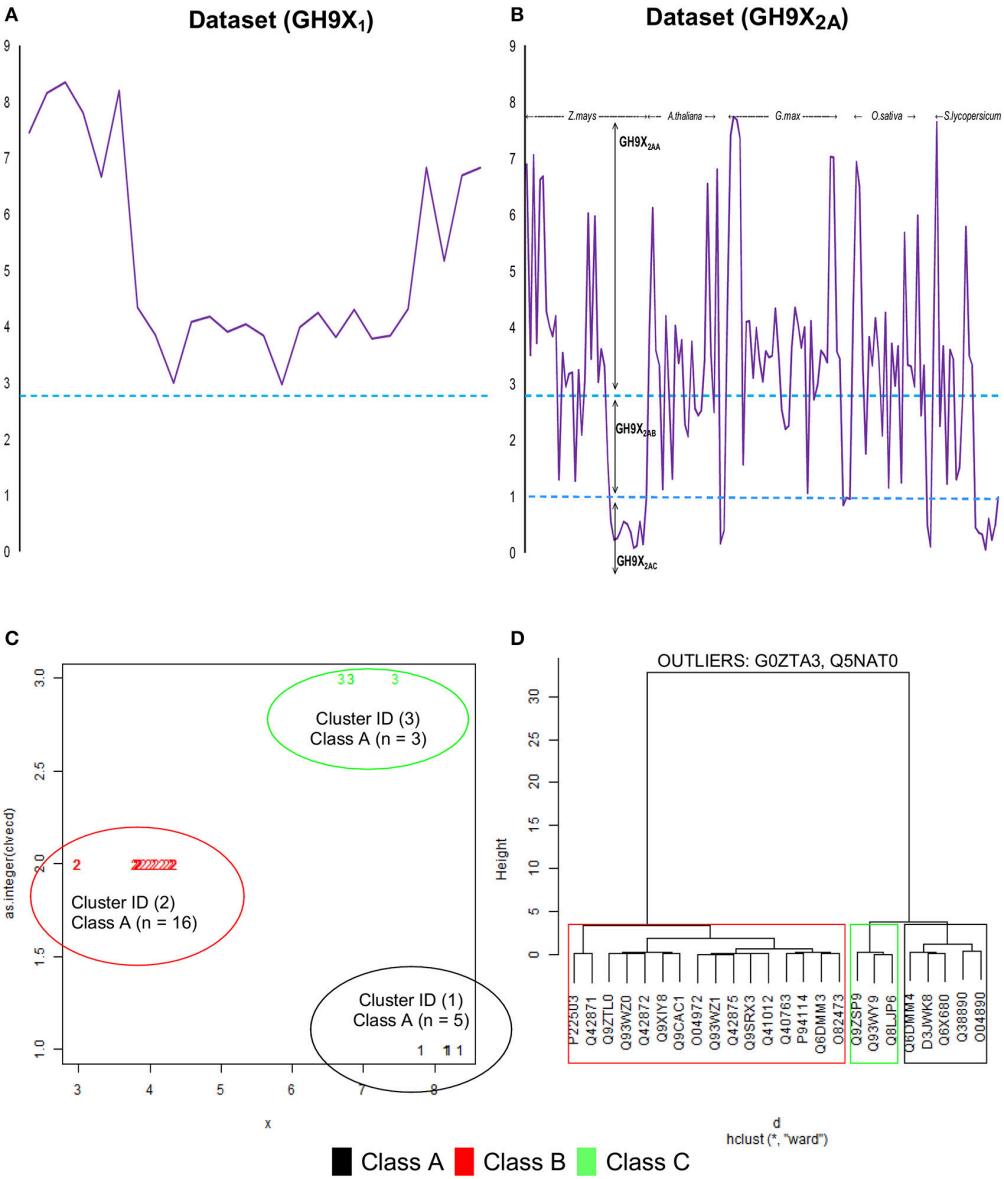
**Computational analysis of datasets GH9X_**1**_ and GH9X_**2*****A***_. (A,B)** Trend of β-values across sequences in representative datasets. Here, β = 2.777, 1.00 and functions to identify sequences with well-spaced interprofile HMM scores (*GH*9*X*_2*AA*_ = 92; *GH*9*X*_2*AB*_ = 28; *GH*9*X*_2*AC*_ = 27) **(C)** k-means clustering of the centroids (**β**) of the clusters that correspond to the previously defined classes A, B, and C of plant GH9 endoglucanases after outlier exclusion (*GH*9*X*_1*A*_ = 24). **(D)** Cluster-dendrogram distribution of sequences of the modified training set (*GH*9*A*_1*A*_ = 5, *GH*9*B*_1*A*_ = 16, *GH*9*C*_1*A*_ = 3).

The computation and selection of the β-values for the training sequences was based on the *in silico* selection of profiles (*HMM*_*GH*9*XI*_) as outlined earlier. Here, *min*(β) ≅ 3.00 (UID, P22503) (Figure 4A and Table S2C), corresponded to a median inter-profile HMM score ≅ 200. Refinement of these computed values was accomplished by analyzing the fluctuations in β-values on *GH*9*X*_2*A*_, and in the intervals ([0, 50), [50, 150), [150, ∞)) (Table 3). The sequence SOLYc05g052530.1.1 had a median interprofile HMM score ≅ 93, and a high β-value (β_*Solyc*05*g*052530.1.1_ ≅ 2.777) (Tables S3B,C). Therefore, (β > 2.777) ∧ (*median* (Δ*HMM*) ≥ 200), was chosen as the major criteria to further partition and evaluate sequences of *GH*9*X*_2*A*_ (*GH*9*X*_2*AA*_ = 92, *GH*9*X*_2*AB*_ = 28, *GH*9*X*_2*AC*_ = 27), and (Figure 4B, Table 3 and Table S3C). The proximity of inter profile HMM (*median* (Δ*HMM*)) scores for sequences with low β-values ((0.67)(*GH*9*X*_2*AC*_) ∈ [0, 50)), whilst the ambiguity of these for sequences with intermediate β-values ((0.3–0.4)_(*GH*9*X*__2*AB*_) ∈ {[0, 50), [50, 150), [150, ∞)}); along with their poor precision and recall precluded their selection as numerical approximations of the threshold value (Table 3 and Table S3C). Conversely, the homogeneity of computed data ((0.86)_(*GH*9*X*__2*AA*_) ∈ [150, ∞)), perfect precision, and high recall, for sequences with β > 2.777, dictated the final value of the filter for shortlisting sequences (*GH*9*X*_2*BA*_) (Table 3, Table S5).

**Table 3:** **Performance of ANN-predictor on *GH*9*X*_2A_**.

**Interval**	**β**	**NSeq**	**M**	**MM**	**NM**	**P**	**R**	**ΔHMM (Observations)**
[0, 50)	β≥2.777	92	92	0	0	100%	85.2%	224.475 (276)
[50, 150)	1.00 ≤ β < 2.777	28	7	8	13	25%	6.5%	133.1 (84)
[150, ∞)	0.00 < β < 1.00	27	9	6	12	33%	8.3%	32.8 (81)

β, Value of statistical filter; Nseq, Number of sequences; M, Number of matches; MM, Number of mismatches; NM, Number of no matches; P, Precision; R, Recall; ΔHMM, Median HMM interprofile difference.

**Figure 5:**
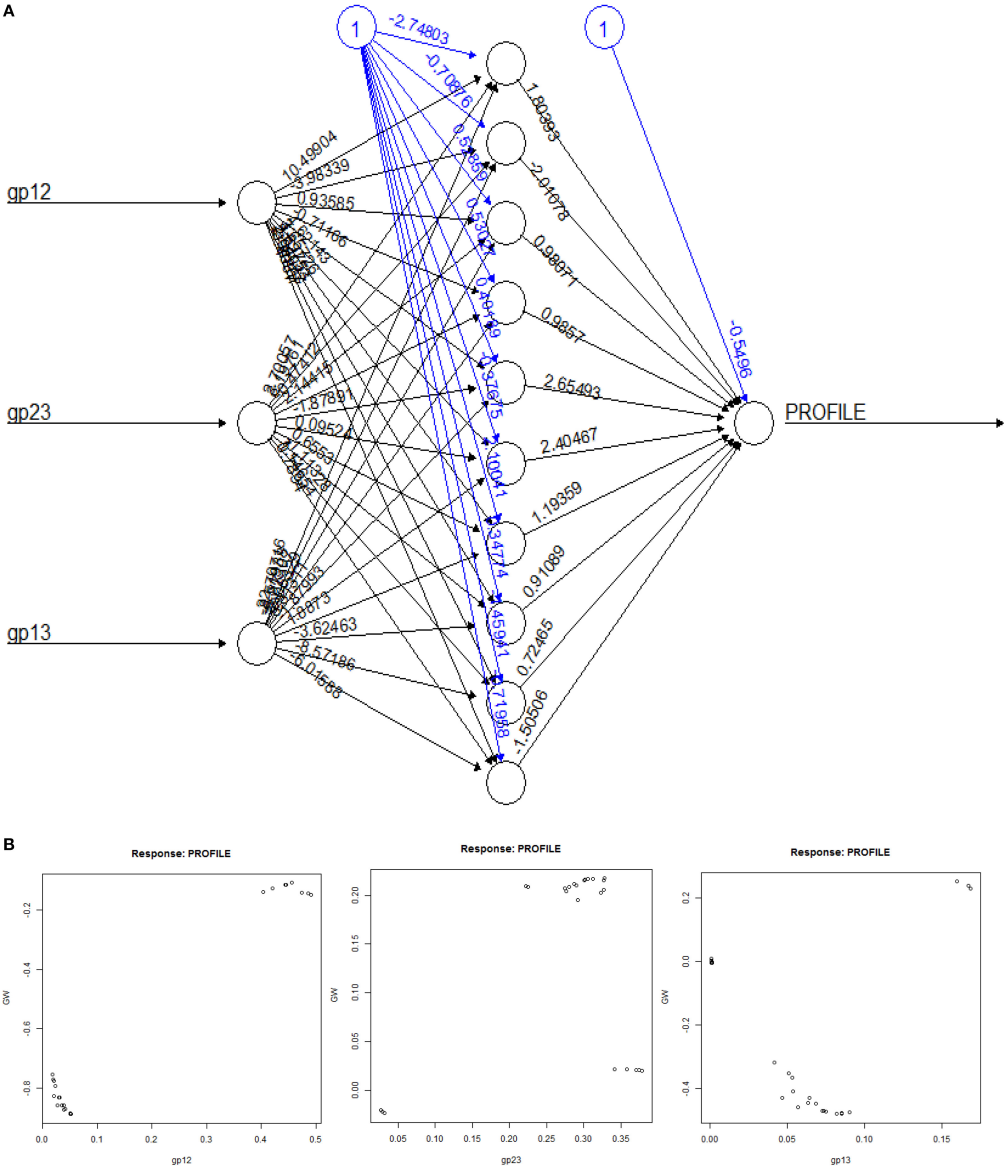
**Details of the predictor-ANN. (A)** The ANN-predicted subclass assignment is dependent on the computed weights and the intercept function. The feedback mechanism for this network is back propagation. **(B)** Plots of α_*ij*_-scores (gp12, gp23, gp13) the associated weights.

We chose the subset (*GH*9*X*_2*AA*_ = 92), since these sequences had a high SNR/widely spaced interprofile HMM scores and therefore possessed adequate class specific discriminatory data (*GH*9*A*_2*AA*_ = 19, *GH*9*B*_2*AA*_ = 43, *GH*9*C*_2*AA*_ = 30). The data from these sequences was, used to a) refine the numerical value of β, and b) define the intervals for the computed ANN scores, used subsequently for unambiguous class assignment (Figure 5; Table S4). The analysis (precision and recall; Table 3) further, served as an index against overestimating the predictions by the ANN on sequences of *GH*9*X*_2*BA*_ (Table 3 and Table S5). The organism specific distribution was: *A. thaliana* (*GH*9*X*_2*AA_atha*_ = 14), *G. max* (*GH*9*X*_2*AA_gmax*_ = 30), *O. sativa* (*GH*9*X*_2*AA_osat*_ = 18), *S. lycopersicum* (*GH*9*X*_2*AA_slyc*_ = 8), and *Z. mays* (*GH*9*X*_2*AA_zmay*_ = 22) (Figure 4B; Table S4). The putative GH9 endoglucanase homologs were identified (*HMM*_*GH*9*X*_; *GH*9*X*_2*B*_), downloaded fom Viridiplantae, filtered (*GH*9*X*_2*BA*_ = 552), and analyzed with the generic class specific HMMs (*HMM*_*GH*9*A*_, *HMM*_*GH*9*B*_, *HMM*_*GH*9*C*_) (Table S5). Interestingly, only five sequences were excluded, despite a seven-order difference in magnitude (*logE*_*GH*9_/*E*_*GH*9*X*_) of the respective *E*-value thresholds.

### Predicted activity of GH9 endoglucanases

The clustered data (β; *GH*9*X*_1_) (Figures 4C,D), was analyzed, wherein, a single cluster-node for each enzyme class was identified $\left({\beta^{\prime }}_{GH9A}≅8.134881;{\beta^{\prime }}_{GH9B}≅\;3.912766;{\beta^{\prime }}_{GH9}≅6.953259;between_{SS}/total_{SS}=0.96\right)$ (Text S4). Two outliers (UIDs G0ZTA3, Q5NAT0) (Figure 4D; Tables S2C,D) were identified, and excluded from further analysis. The class specific cluster means was approximated by the group scores of each training sequence $\left(\beta^{\prime }\sim \alpha_{12}+\alpha_{23}+\alpha_{13};\;{GH9X}_{1A}=24\right)$. The ANN scores (β″), thus computed (leave-one-out) when compared with the cluster means (χ^2^ = 0.005; *df* = 23; *p* = 0.001) (Figure 5; Table S2D), clearly suggest the equivalence of the ANN predictor with the dominant enzyme function of the sequence of interest (Equations 3–5; ANN prediction ≅ cluster mean of a class ≅ max$\left({\bar{HMM}}_{GH9A},{\bar{HMM}}_{GH9B},{\bar{HMM}}_{GH9C}\right)$. The above criteria was used on *GH*9*X*_2*AA*_ (χ^2^ = 0; *df* = 91) to associate, dominant enzyme function with statistically defined class specific intervals of the ANN-predictors $\left(\beta_{GH9A}^{\prime \prime },\beta_{GH9B}^{\prime \prime },\beta_{GH9C}^{\prime \prime }\right)$ and α_*ij*_-values (Table 4 and Table S4).

**Table 4:** Bounds and conditions for class assignment of GH9 endoglucanases^*^.

**Enzyme**	**Rules**	
Class A	((9.95 < β*′′_GH_*_9_*_A_* < 10.779)∧(α13 < 0.02))	
Class B	High	3.45 < β*′′_GH_*_9_*_B_* < 5.55
	Low	5.55 ≤ β*′′_GH_*_9_*_B_* < 8.2052
Class C	$\begin{array}{l} \left(8.2052\leq \beta_{GH9C}^{\prime \prime }\leq 9.95\right)\vee \left((9.95<\beta_{GH9C}^{\prime \prime }<10.779\right)\wedge \\ \left(\alpha_{13}>0.02))\vee (10.779\leq \beta_{GH9C}^{\prime \prime }\leq 11.371\right) \end{array}$	

* Dataset used for this definition was GH9X_2AA_.

$\beta_{GH\text{9}A}^{\prime \prime },\;\beta_{GH\text{9}B}^{\prime \prime },\;\beta_{GH\text{9}C}^{\prime \prime }:=$ ANN-predicted values for filtered sequences of (GH9X_2AA_ = 92).

**Figure 6:**
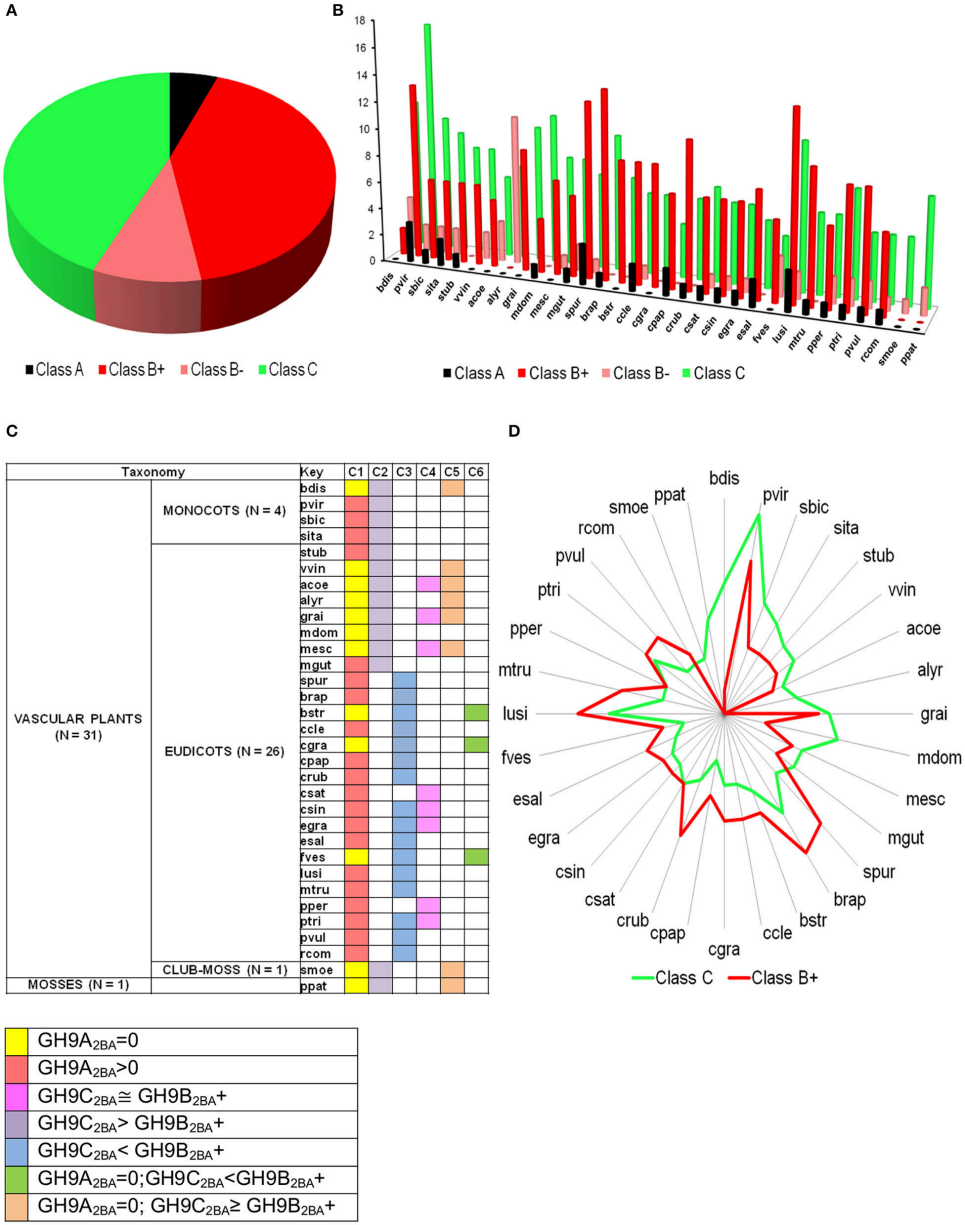
**Prediction profile of GH9 endoglucanases in test dataset (GH9X_**2*****BA***_ = 552). (A)** Generic distribution of sub-class members in the primary proteomes of 32 green plants with sequenced genomes. Here, class B+ refers to sequences with high-confidence in their assignment, in contrast with class B− data. **(B)** Taxonomic spread of classes A, B, and C in selected phytozome data, **(C)** Association table of class specific GH9 proteins, and **(D)** Radar plot of the distribution of members of classes-B+ and C.

The bounds, thus defined, were then used to stratify the scores of the test sequences in *GH*9*X*_2*BA*_ (Tables 4, 5 and Table S5). Our results suggest the following taxonomic distribution of GH9 proteins (*GH*9*A*_2*BA*_ ≈ 6%, *GH*9*B*_2*BA*_ ≈ 50%, *GH*9*C*_2*BA*_ ≈ 44%) (Figures 6A,B). Additional findings include *GH*9*B*_2*BA*_ ≅ *GH*9*C*_2*BA*_, *GH*9*A*_2*BA*_ ≅ *GH*9*C*_2*BA*_, *GH*9*C*_2*BA*_ > *GH*9*B*_2*BA*_, *GH*9*B*_2*BA*_ > (2)(*GH*9*C*_2*BA*_) ≅ *GH*9*A*_2*BA*_ (Table 5 and Figures 6C,D). The following gene(s) of *O. sativa* (LOC_Os02g05744, LOC_Os09g23084, LOC_Os02g50490, LOC_Os08g32940) and *A. thaliana* (AT2G32990) were reannotated by our ANN-predictor as class C (Buchanan et al., 2012; Xie et al., 2013). The sequences, annotated by our prediction scheme $\left({GH9A}_{2BA}=31,{{GH9B}^{+}}_{2BA}=231,{{GH9B}^{-}}_{2BA}=49,{GH9C}_{2BA}=241\right)$ are available in fasta format (Text S6–S9).

**Table 5:** **Sub-class prediction and distribution of GH9 endoglucanases in Viridiplantae**.

**S. No**.	**Organism (key)**	**GH9 members^$^**	Sub-class^*^	(**β** > 2.777)				
				**GH9A_2BA_**	**GH9B**_2BA_		**GH9C_2BA_**	**Total**
					**High**	**Low**		
1.	*Aquilegia coerulea* (acoe)	23	22	0	5	3	6	14
2.	*Arabidopsis lyrata* (alyr)	25	25	0	0	11	7	18
3.	*Arabidopsis thaliana*^#^ (atha)	26	26	2	8	0	4	14
4.	*Brassica rapa* FPsc (brap)	39	39	1	14	0	10	25
5.	*Brachypodium distachyon* (bdis)	23	23	0	2	4	11	17
6.	*Boechera stricta* (bstr)	23	23	0	9	0	7	16
7.	*Citrus clementina* (ccle)	24	24	2	9	1	6	18
8.	*Capsella grandiflora* (cgra)	23	23	0	9	0	6	15
9.	*Carica papaya* (cpap)	20	20	2	7	0	4	13
10.	*Capsella rubella* (crub)	24	24	1	11	0	6	18
11.	*Cucumis sativus* (csat)	19	19	1	7	1	7	16
12.	*Citrus sinensis* (csin)	23	23	1	7	1	6	15
13.	*Eucalyptus grandis* (egra)	29	29	1	7	1	6	15
14.	*Eutrema salsugineum* (esal)	24	22	2	8	0	5	22
15.	*Fragaria vesca* x h (fves)	23	22	0	6	3	4	13
16.	*Glycine max*^#^ (gmax)	40	39	5	17	0	8	30
17.	*Gossypium raimondii* (grai)	26	26	0	9	0	10	19
18.	*Linum usitatissimum* (lusi)	45	45	3	14	2	11	30
19.	*Malus domestica* (mdom)	53	53	1	4	0	11	16
20.	*Manihot esculenta* (mesc)	28	28	0	7	1	8	16
21.	*Mimulus guttatus* (mgut)	23	23	1	6	2	8	17
22.	*Medicago truncatula* (mtru)	32	32	1	10	0	6	17
23.	*Oryza sativa*^#^ (osat)	26	26	3	8	0	7	18
24.	*Physcomitrella patens* (ppat)	21	21	0	0	2	8	10
25.	*Prunus persica* (pper)	19	19	1	6	2	6	15
26.	*Populus trichocarpa* (ptri)	32	32	1	9	2	8	20
27.	*Panicum virgatum* (pvir)	50	50	3	13	2	17	35
28.	*Phaseolus vulgaris* (pvul)	23	23	1	9	2	5	17
29.	*Ricinus communis* (rcom)	21	21	1	6	1	5	13
30.	*Sorghum bicolor* (sbic)	25	25	1	6	2	10	19
31.	*Setaria italica* (sita)	25	25	2	6	2	9	19
32.	*Selaginella moellendorfii* (smoe)	14	14	0	0	1	5	6
33.	*Salix purpurea* (spur)	34	34	3	13	1	7	24
34.	*Solanum lycopersicum*^#^ (slyc)	22	22	2	2	0	4	8
35.	*Solanum tuberosum* (stub)	24	24	1	6	0	8	15
36.	*Vitis vinifera* (vvin)	23	22	0	6	2	8	16
37.	*Zea mays*^#^ (zmay)	38	38	7	8	0	7	22

# Reference sequences (N_2_).

$ Selected Phytozome v10.3 sequences were searched using the E5xyD subset of HMMs (E_GH9X_ = 10).

* GH9-homologs were then searched using the E4xyD subset of HMMs (E_GH9A_ = E_GH9B_ = E_GH9C_ = 10E-06). The class assignment corresponded to the highest average sequence score with the profile-HMMs.

xh, Hybrid.

High, Low: Confidence in class B assignment (Table 2).

### Assessment of predictor performance

The *in silico* HMM profiles for the training sequences when assessed, as defined (Defs.1–3, 10–12), using the LOOCV, had a precision of 100% (*GH*9*X*_1_ = 26) (Tables S3A,B). The precision of the ANN computed approximations of the cluster means, and using the LOOCV, on the training set after outlier exclusion (*GH*9*X*_1*A*_ = 24) was 100% when assessed by the aforementioned criteria (Equation 5). Overestimation by our HMM-ANN pipeline was examined by the performance of these predictions on *GH*9*X*_2*A*_ (Table 3).

### Meta-analysis and significance of the ANN-based prediction schema

**Figure 7:**
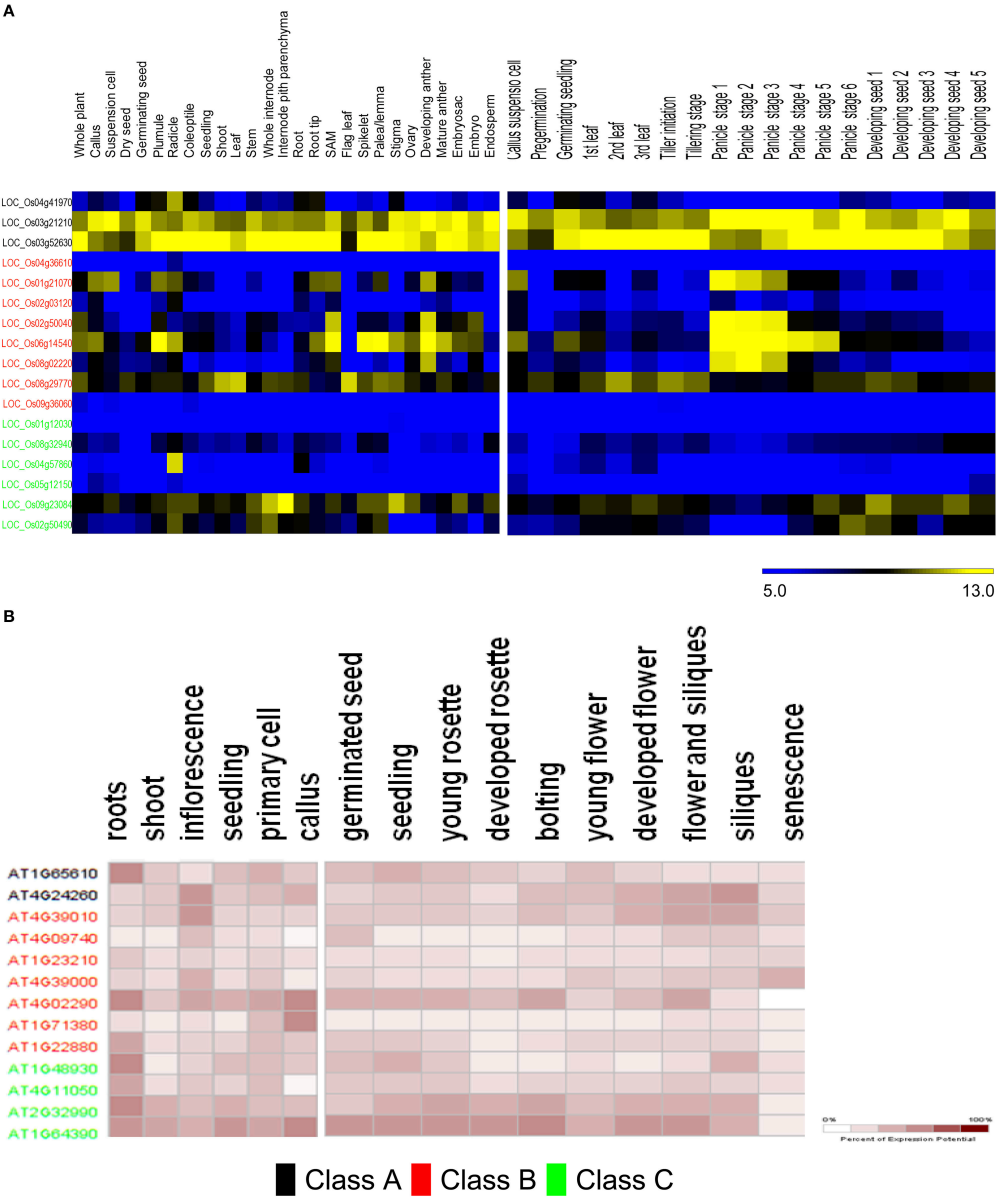
**Meta-analysis of expression data of ANN-predicted GH9 endoglucanses**. Heat maps showing expression patterns of classes A, B, and C in *O. sativa* **(A)** and *A. thaliana* **(B)** in anatomical tissues and at various stages of development. The stages/tissues have been marked at the top. The color bar for *O. sativa* **(A)** represents log2 expression values, blue indicating low-level expression, black medium, and yellow high-level expression, while the same for *A. thaliana* **(B)**, is a gradient from white to maroon and represents percentage of expression potential at the right corner. The downloaded high-resolution images were adapted and presented as such.

The predicted distribution of rice GH9 endoglucanases (*GH*9*A*_2*AA*−*osat*_ = 3, *GH*9*B*_2*AA_osat*_ = 8, *GH*9*C*_2*AA_osat*_ = 7) was examined across several tissues (Table S6). Most putative enzymes with predicted class C activity express poorly or not at all (Figure 7A). Exceptionally, LOC_Os04g57860 has very high expression in the radicle, with minimal expression in other tissues studied. LOC_Os09g23084 and LOC_Os02g50490, in contrast have a broad expression pattern, with maximum levels of LOC_Os09g23084 mRNA levels observed in the internode pith parenchyma, whole internode, and stigma. The mRNA distribution of the class B endoglucanases (*GH*9*A*_2*AA_atha*_ = 2, *GH*9*B*_2*AA_atha*_ = 8, *GH*9*C*_2*AA_atha*_ = 4) is evenly spread with maximum expression in the developing anthers and shoot apical meristem (Figure 7A). LOC_Os06g14540, exhibits the highest expression in the plumule, shoot apical meristem, spikelet, palea/lemma, and the developing anthers. LOC_Os04g36610 and LOC_Os09g36060 exhibited very low expression minimal mRNA levels in all the tissues analyzed.

The expression of class B and C genes was also studied in various developmental stages (Figure 7A). The transcripts of two class C putative genes LOC_Os09g23084 and LOC_Os02g50490, were mainly detected in the leaf (stages −1, −3), panicle (stages −5, −6), and seed developmental stages (stages 1–4). The remaining class C genes do not seem to express at the levels detected in the developmental stages analyzed. Most of the class B enzymes, except (LOC_Os04g36610, LOC_Os02g50040, and LOC_Os09g36060) on the contrary, exhibit developmental stage-specific expression pattern. The transcripts of LOC_Os06g14540, LOC_Os01g21070, LOC_Os02g50040, and LOC_Os08g02220 accumulate in the early stages of panicle development, whereas, LOC_Os06g14540, LOC_Os01g21070 were mainly detected in the callus suspension, LOC_Os08g29770 and LOC_Os06g14540 mainly express during pre-germination, and the germinating seed stages, respectively. LOC_Os08g29770, has reasonably high mRNA levels during leaf development (stages 1–3), tiller initiation, tillering, and the seed stages (stages 2, 3; Figure 7A). The data for *A. thaliana* (Figure 7B) suggests that predicted class C genes have similar levels of expression in the anatomical tissues examined, with the loci AT2G32990 and AT1G64390 exhibiting high mRNA levels in the callus, seedling, inflorescence, cell culture, shoot, and root. Class B genes, in contrast, have a poor expression pattern, with the only exceptions being AT4G02290. The stages of maximal gene expression coincide with the stages of callus (AT4G02290 and AT1G71380), inflorescence (AT4G39010, AT4G09740, AT4G39000, and AT4G02290), and root tissues (AT4G02290 andAT1G22880; Figure 7B).The expression patterns also suggest that, AT2G32990 and AT1G64390 (class C); AT4G39010 and AT4G02290 (class B), seem to play an important role at all stages of *A. thaliana* development. The class B locus, AT4G39000 exhibits high expression during senescence (Figure 7B).

## Discussion

### Unambiguous assertion of classes A, B, and C in GH9 endoglucanses

We have utilized empirical data to identify novel and uncharacterized GH9 endoglucanases from Viridiplantae. The utility of substrates and/or reaction chemistry, structural data, and transcript information to cluster enzymes has been attempted in earlier work, *albeit*, in different biological systems (Kundu, 2012). Since, biochemical information for these enzymes is sparse, we combined available data with well-grounded analytic methods (Figure 2) to predict dominant function in GH9 endoglucanases. A general binary classification schema, using a round robin format will convert n-loci into $C_{2}^{n}$ pairs, score each, and poll the votes to achieve an overall dominant class (Savicky and Furnkranz, 2003). Since, the unambiguous identification of class C enzymes, mandates, the sequestration of their raw HMM scores, the variance between data pairs, was computed. The ANN prediction was based on the pattern of computed weights for α_*ij*_(= *gp*_*ij*_) and its equivalence with the cluster mean (Equations 3-5) for each sequence of the training set. Equation 2, may be written as:

(6) $$\alpha_{ij}=gp_{ij}={\left(\left|\mu_{i}-\mu_{j}\right|\right)}^{2}/\left(100\right)(\sqrt[{2}]{\left(\sigma_{i}^{2}\;+\;\sigma_{j}^{2}\right)/\tau })$$

(7) $$\alpha_{ij}\propto 1/\sqrt{\bar{\sigma_{ij}^{2}}}$$

Clearly, the proportionality constant (γ_*ij*_) for Equation 7, can function as a multiplier (1 < γ_*ij*_ < ∞) or as a divisor (0 < γ_*ij*_ < 1). This modification, compensates for the inverse relation between the α_*ij*_-values and the average variance of the relevant data points. Further:

(8) $$∵\beta =\sum \alpha_{ij}=\sum \gamma_{ij}/\sqrt{{\bar{\sigma }}_{ij}^{2}}$$

It follows, then, that as the difference between the raw HMM profile scores (Δ*HMM*) increases, the corresponding β-value is incremented. The ambiguity in the data trend observed for *GH*9*X*_2*A*_ (Table 3) can be interpreted in terms of the inter profile HMM differences (Δ*HMM*). Thus, for the set (*GH*9*X*_2*AC*_; β < 1.00), ~67% of the data was sequestered in the interval [0, 50) or (0.67)({(*median*(Δ*HMM*_*GH*9*AB*_) ∧ *median*(Δ*HMM*_*GH*9*BC*_) ∧ *median*(Δ*HMM*_*GH*9*AC*_)}) < 50. Similarly, for β > 2.777, ≈ 86% of the data in *GH*9*X*_2*AA*_ belonged to the interval [150, ∞) or (0.86)({(*median*(Δ*HMM*_*GH*9*AB*_) ∧ *median*(Δ*HMM*_*GH*9*BC*_) ∧ *median*(Δ*HMM*_*GH*9*AC*_)}) ≥ 150. However, for intermediate values of β(1.00 ≤ β < 2.78 this data, for the highest scoring subset *GH*9*X*_2*AB*_, is heterogeneous, ambiguous, and spread uniformly (≈ 30–40%) across all the intervals examined (Table 3 and Table S3C). This data further vindicated our choice of the threshold value.

A comparison with previous predictions of cellulase activity suggests interesting differences. Whilst, sequences with purported class A activity, coincided with earlier work, our annotation attributes dominant class C catalytic activity to a majority of the remainder $\left({GH9C}_{2BA}>{GH9B}_{2BA}^{+}\right)$ (Table 5). This surprising finding, is in complete contrast to earlier predictions, wherein class B enzymes predominate, i.e., *GH*9*B* ≫ *GH*9*C* (Montanier et al., 2010; Buchanan et al., 2012; Xie et al., 2013). Since, the selection of these sequences is threshold driven, the inclusion of sequences with low confidence scores $\left({GH9B}_{2BA}^{-}=49\right)$ (Table 5 and Figure 6A) was taken into consideration for some of these calculations. However, despite this, i.e., the total class B enzymes are marginally higher than class C members $\left({GH9B}_{2BA}^{+}+{GH9B}_{2BA}^{-}>{GH9C}_{2BA};\;50\%\text{ vs}.44\%\right)$. In earlier studies, the overwhelming bias (*GH*9*B* ≅ (5)(*GH*9*C*)), may be attributed to the indices chosen (sequence similarity and their modifications) to cluster *A. thaliana* and *O. sativa* data (Montanier et al., 2010; Xie et al., 2013). Additionally, since later work on other organisms (*Populus* spp., *Hordeum vulgare, Z. Mays, Sorghum bicolor, Brachypodium distachyon*) used this data as a classification schema, the results were similar (Buchanan et al., 2012; Xie et al., 2013). In our analysis, considerable emphasis has been given to the correlation between the function and organization of class specific sequence or 3D modeled data such as the presence or absence, mutagenesis, and biochemistry of specific regions (secretory peptide, trans-membrane, CBM49; Figure 3C) in characterized proteins. The resultant class-specific pHMMs with stringent inclusion thresholds, filters, and clustering algorithms, are thus, able to generate noise-free data (distinguish higher- and lower-scoring regions of a particular sequence); thereby generating non conflicting predictions of class A, B, and C enzyme activity (Table S5).

### Function of GH9 endoglucanases may vary with cellulose content

The role of GH9 endoglucanses in green algae is not clear, and may range from facilitating intercellular/cell matrix adhesion (hydrolysis of cell wall cellulose when present), to extracellular digestion and assimilation of nutrients. Whilst, the presence of cellulose, as a component of the extracellular matrix/cell wall in *Chlorella* (order Trebouxiophyceae), *Oedogonium* (order Chlorophyceae), *Bryopsis* spp. (order Ulvophyceae;), and *C. corallina* (order Charophyceae;) suggest the former; the latter may prevail even in the absence of cell wall cellulose (*C. reinhardtii*, order Chlorophyceae; *O. lucimarinus*, and *M. pussila* spp., order Prasinophyceae; Becker et al., 1989; Estevez et al., 2008; Domozych et al., 2009, 2012; Rodrigues and da Silva Bon, 2011; Blifernez-Klassen et al., 2012; Ciancia et al., 2012). Our results, too, using the generic-HMM (*HMM*_*GH*9*X*_) ignore putative GH9 endoglucanases, from *O. lucimarinus* and *M. pussila* spp. We also observed that green algae spp., descending from *Chlamydomonas* spp. or *Volvox* spp. (*C. reinhardtii, C. subellipsoidea, V. carteri*), despite being selected, by the generic and class specific pHMMs (*HMM*_*GH*9*X*_, *HMM*_*GH*9*A*_, *HMM*_*GH*9*B*_, *HMM*_*GH*9*C*_), had β-values < 1.00. The proximity of HMM scores (*median*(Δ*HMM*) < 25) (Table S5), too, suggests an overlapping functionality with conflicting biological relevance, thereby, justifying their exclusion.

### Taxonomic distribution and expression pattern of GH9 endoglucanase may dictate dominant function

The predicted TM domain present in Class A GH9 endoglucanases localizes these enzymes to the membranous compartments of the cell. This suggests that the cellulase activity of this sub-class may be critical to cellulose assembly. In particular, the contribution of this subclass to the formation of a cellulosome, as a protein-carbohydrate connector is, well characterized (Mansoori et al., 2014). Yet, another complementary role for these enzymes is the utilization of the oligosaccharide generated, as a primer. These critical processes in the cellulose based-tiling of the cell wall, clearly are dependent on the focal presence of these endoglucanases. In a related study, class A enzymes were participants in cytokinesis, as well (Zuo et al., 2000). The absence, therefore, of this subclass, in some genera may be expected to retard the biochemical machinery involved in the breakdown of the primary cell wall. Development of uninterrupted xylem cells (absence of cytokinesis), too, could facilitate this. We noticed that a complete absence of class A enzymes (≈ 47.6%), also, interestingly, coincided with increased numbers of class C GH9 endoglucanases in a large number of green plants (≈ 70%; Figure 6C). This includes some of the grasses, and other herbaceous plants.

In graminaceae, class C sequences clearly predominate or are at most approximately equal $\left({GH9C}_{2BA}\geq {GH9B}_{2BA}^{+}\right)$ (Figures 6C,D). These sequences, at least, hypothetically appear to be more efficient (possess a broader substrate range) as catalysts, a factor which may impede the development of a secondary cell wall, as well as render the primary structure pliable and responsive to abiotic stressors (Kundu, 2015a). Other herbaceous plants such as *V. vinifera, P. patens, S. moellendorffii*, too, possess a non-woody stem, perhaps, secondary to heightened cellulase class C activity. The whole internode, internode pith parenchyma, and developing seed are rich in cellulose content, a factor that may highlight the observations of high mRNA levels in the precursor stem (*O. sativa*; LOC_Os09g23084, LOC_Os02g50490) or shoot (*A. thaliana*; AT2G32990, AT1G64390) regions (Figure 7). The abundance of cellulose in some of these tissues suggests, that despite the improved substrate range (crystalline, amorphous), zero order kinetics may predominate in these. This would imply, that putative class C enzymes possess a high Km value, which would render them ineffective when the cellulose content of tissues is minimal (flower, senescence). The reduced cellulose content of the inflorescence and senescent stages (*A. thaliana*) or developing leaf and panicle (*O. sativa*), may require the activities of a biochemically more efficient enzyme (lower Km) with the consequent first order kinetics. Class B enzymes may fulfill this role *in vivo*. Elevated mRNA expression levels of class B genes during these stages in both, of *O. sativa* and *A. thaliana* could support this notion (Figures 7A,B).

### Molecular evolution of classes B and C enzymes may reflect the development of complex physiology

Our findings suggest that the number of GH9 endoglucanases of class B varies inversely with class C enzymes. The non-woody stem of simpler plants suggests a less complex genome organization, thereby, signifying a reduced proteome with reduced differentiation. As green plants evolved, they incorporated genome segments that coded for proteins of greater complexity and broader functions, the need for an efficient cellulase waned. Thus, class B enzymes appear to frequent the woody, longer living, and more specialized plants, allowing fully developed primary and secondary cell wall structures. The class C identifier, is the CBM49, a 100-120 amino acid region rich in the aromatic and bulky Tryptophan/Tyrosine/Phenylalanine residues. This module is present at the C-terminal end, and is linked by a short stretch of amino acids to the remainder of the protein, which is, in fact, a *de facto* class B sequence (Figure 3C; Urbanowicz et al., 2007b). It is possible that, with evolution, this region was spliced out during transcript processing, resulting in the reduced contribution of class C enzymes to general plant physiology, with a reciprocal, dominant presence of class B sequences. Nevertheless, the broad substrate range might compensate, for the poor distribution and/or catalytic efficiency (high Km), thereby, conferring an evolutionary advantage to plants with a functional set of class C GH9 endoglucanases.

### Scope and limitations of *in silico* classification of GH9 endoglucanases

Whilst, accurate, the strength of the assertion of subclass assignment, is dependent on the availability of empirical data (train and validate the HMMs and the ANN), stringency of the sequence filter, and noise-free data (high Signal-to-noise ratio). Clearly, these restrict the utility of the HMM-ANN predictions as a general purpose annotator. Additionally, there is also an in increased loss of information, in terms of sequence(s) elimination (~7 vs. ~37%). The integrated pipeline, is also, unlikely to benefit workers with poly-functional enzymes such as the superfamilies of heme-dependent mono- and 2-oxoglutarate dependent di-oxygenases (*N*_*pHMM*_ > 25) (Kundu, 2012, 2015b). Thus, the enzymes anthocyanidin synthase (EC 1.14.11.19) and clavaminate synthase (EC 1.14.11.21) catalyze substrate hydroxylation and desaturation in tandem, and could, contain overlapping generic 2OG-dependent, hydroxylation, and desaturase profiles. Nevertheless, here too, the subfamilies of the mono-catalytic proline 3- and 4-hydroylases (EC 1.14.11.28, EC 1.14.11.7; EC 1.14.11.2), pentalenolactone- and phytanoic acid-hydroxylases (EC 1.14.11.36; EC 1.14.11.18, might constitute suitable candidates for automatic functional assignment. Since, the HMM-ANN pipeline is dependent on empirical evidence of known function(s), this method may not be suitable as a general sequence annotator.

## Concluding remarks

Cellulose digesting GH9 endoglucanases, potentially, have roles in modifying the anatomy and the physiology of plants. The influence on development and response to stress, mandates the pre-emptive breakdown of this glucan. The emergence of plant biomass as a source of biofuel, too, may benefit from the identification of cellulases with a broader substrate range. Alternately, *in vivo* modification could result in plants with abundant and accessible precursor material, facilitating germination, growth, and development. A role for these versatile enzymes, as part of the microbiome promoting biofilms, too, could influence our comprehension of favorable biota for optimal cultivation conditions. However, the limited biochemical characterization of plant proteins (structure, kinetic, mutagenesis), complexity of genetic modification protocols, susceptibility to biotic and abiotic stressors, and heterogeneous growth even in controlled environments, all exert contributory offsets to smooth implementation of these ideas. Despite these, the use of next-generation sequencing, with its precursor genomic data and putative proteome, has the potential to accelerate *in vitro* characterization of computationally predicted functional modules in hypothetical ORFs of plant genomes. Our work, attempts to bridge this divide by constructing a publically available repository of high quality class C plant GH9 endoglucanase sequences.

## Author contributions

SK outlined and designed the study, designed the algorithm for prediction, manually collated all the sequences and their references, carried out the computational analysis, constructed the models, formulated the filters, wrote all necessary code, and the manuscript. RS outlined the study, and participated in revising the manuscript.

### Conflict of interest statement

The authors declare that the research was conducted in the absence of any commercial or financial relationships that could be construed as a potential conflict of interest.

## Abbreviations

ANN: Artificial Neural Network
CBM: Carbohydrate Binding Module
GH: Glycoside Hydrolase
HMM: Hidden Markov Model
UID: Uniprot Identifier.
